# Multicolor banding remains an important adjunct to array CGH and conventional karyotyping

**DOI:** 10.1186/1755-8166-6-55

**Published:** 2013-12-05

**Authors:** Susan M Bint, Angela F Davies, Caroline Mackie Ogilvie

**Affiliations:** 1 Cytogenetics department, GSTS-Pathology, Guy’s and St. Thomas’ Hospital NHS Foundation Trust, London SE1 9RT, UK; 2 Genetics Centre, Guy’s and St Thomas’ NHS Foundation Trust, London SE1 9RT, UK; 3 Division of Genetics and Molecular Medicine, King’s College London, School of Medicine at Guy’s, King’s College and St Thomas’ Hospitals, London SE1 9RT, UK

**Keywords:** Multicolor banding, Fluorescence *in situ* hybridization, Array CGH, Karyotype analysis, Complex chromosome rearrangements

## Abstract

**Background:**

Array comparative genomic hybridization (CGH) for high resolution detection of chromosome imbalance, and karyotype analysis using G-banded chromosomes for detection of chromosome rearrangements, provide a powerful diagnostic armoury for clinical cytogenetics. However, abnormalities detected by karyotype analysis cannot always be characterised by scrutinising the G-banded pattern alone, and imbalance detected by array CGH cannot always be visualised in the context of metaphase chromosomes. In some cases further techniques are needed for detailed characterisation of chromosomal abnormalities. We investigated seven cases involving structural chromosome rearrangements detected by karyotype analysis, and one case where imbalance was primarily detected by array CGH. Multicolor banding (MCB) was used in all cases and proved invaluable in understanding the detailed structure of the abnormalities.

**Findings:**

Karyotype analysis detected structural chromosome rearrangements in 7 cases and MCB was used to help refine the karyotype for each case. Array CGH detected imbalance in an eighth case, where previously, G-banded chromosome analysis had reported a normal karyotype. Karyotype analysis of a second tissue type revealed this abnormality in mosaic form; however, MCB was needed in order to characterise this rearrangement. MCB provided information for the delineation of small deletions, duplications, insertions and inversions and helped to assign breakpoints which were difficult to identify from G-banded preparations due to ambiguous banding patterns.

**Conclusion:**

Despite the recent advance of array CGH in molecular cytogenetics we conclude that fluorescence *in situ* hybridization, including MCB, is still required for the elucidation of structural chromosome rearrangements, and remains an essential adjunct in modern diagnostic laboratories.

## Background

Multicolor banding (MCB) is a highly reliable and reproducible fluorescence *in situ* hybridization (FISH) technique that can be used for the detection of balanced and unbalanced chromosome rearrangements and to help characterize complex chromosome rearrangements and marker chromosomes [1,2] that cannot be characterized by array comparative genomic hybridization (CGH) or by G-banded chromosome analysis and standard FISH techniques alone. Images are processed using specialised software (MetaSystems - *Isis*) which converts the fluorescence profiles along the length of the chromosome to pseudocolored bands [1,3]. These pseudocolored bands can then be compared to standard G-banded ideograms [4]. The *Isis* software enables selection of the number of pseudocolored bands on the chromosome to be analysed; it is therefore possible to achieve higher resolution with MCB than that using standard G-banding [3,5].

Array CGH has replaced karyotype analysis as a “first line” genetic test for most referral indications in many laboratories [6-8]; however, it cannot determine the position of rearranged segments within the genome, or provide information on balanced rearrangements. Full characterisation of chromosome abnormalities is important in order to establish genotype-phenotype correlation, provide prognoses, and estimate reproductive risks for the proband and the family.

We describe 8 cases where MCB has been used as an adjunct to karyotype analysis, routine FISH analysis and array CGH in order to characterise structural chromosome aberrations.

## Results

### Case 1

#### Referral reasons

Age 4 months - Hypotonia, obesity, developmental delay, mild/moderate MR and suspicion of Prader-Willi syndrome.

Age 16 years - no reading & writing, simple speech, long face, right sided convergent squint, long tapering fingers, large hands and feet and overcrowded teeth.

#### Preliminary karyotype

46,XX,12p+

#### Investigations

Subtelomere Multiplex Ligation-dependent Probe Amplification (MLPA);

Fluorescence in situ hybridization (FISH) with a locus-specific probe for a locus within band 12p13.2;

MCB probe set for chromosome 12 (XCyte 12).

MLPA, using probes specific for loci within the short and long arm subtelomere regions of every chromosome, showed a deletion of two different probes for the subtelomere region of the chromosome 12 short arm (0.170Mb and 0.287Mb) from the telomere. No duplication or deletion of any other subtelomere region was apparent.

**Figure 1:**
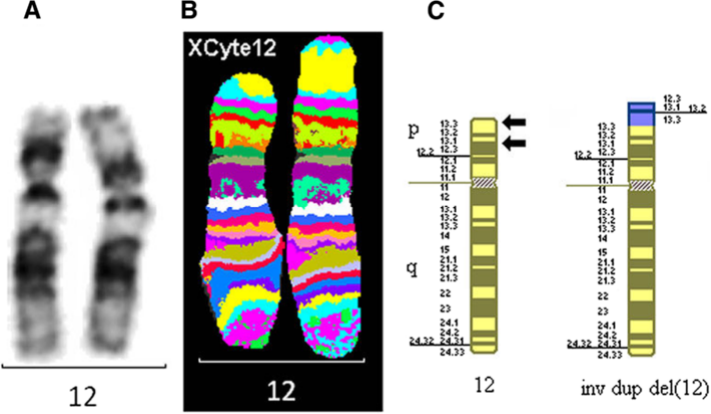
**Chromosome 12s for case 1.** G-banded chromosome 12s **(A)**, multicolor banded chromosome 12s **(B)** and partial G-banded ideogram **(C)** for case 1. Arrows denote breakpoints.

The MCB pattern on the abnormal chromosome 12 was informative for the band order and indicated an inverted duplication of the terminal region of the short arm (Figure 1).

#### Final karyotype

46,XX,inv dup del(12)(qter→p13.3::p13.3→p12.3:) [9-11]

### Case 2

#### Referral reasons

Age 8 months - axial hypotonia and delay in motor milestones.

#### Preliminary karyotype

46,XX,der(7)[39]/46,XX[13]

#### Investigations

Subtelomere FISH with probes specific for the short and long arm subtelomere regions of chromosome 7;

Locus-specific probe FISH;

MCB probe set for chromosome 7 (XCyte 7).

**Figure 2:**
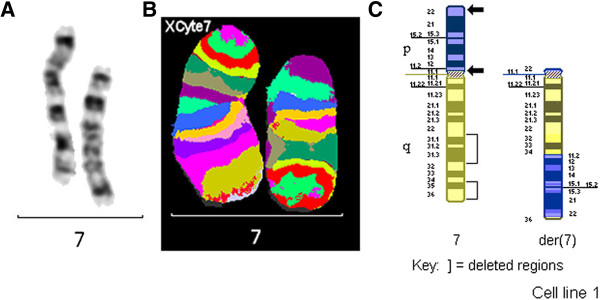
**Chromosome 7s for case 2.** G-banded chromosome 7s **(A)**, multicolor banded chromosome 7s **(B)** and partial G-banded ideogram **(C)** for case 2. Please note that the arms of chromosome 7 are depicted in different colours, for clarity. Arrows denote breakpoints.

The MCB pattern on the abnormal chromosome 7 was informative for the band order, and showed a complex rearrangement involving an insertion event and a deletion of the regions 7q22→7q32 and 7q34→7q36 (Figure 2).

#### Final karyotype

46,XX,der(7)(pter→p22::p11.2→q22::q32→q34::p11.2→p22::q36→qter)[39]/46,XX[13].

### Case 3

#### Referral reasons

Age 1 month - cleft lip & palate, hypotonia, depressed nasal bridge, flat occiput, hypoglycaemia and polyhydramnios in pregnancy.

#### Preliminary karyotype

46,XX,add(7)(q22)

#### Investigations

Whole chromosome paint specific for chromosome 7 (WCP7);

Partial chromosome paints specific for the short arm and long arm of chromosome 7 (PCP7p, PCP7q).

#### Microsatellite analysis

MCB probe set for chromosome 7 (XCyte 7).

**Figure 3:**
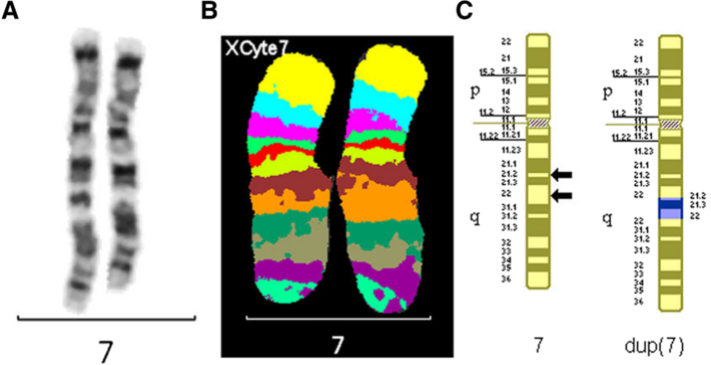
**Chromosome 7s for case 3.** G-banded chromosome 7s **(A)**, multicolor banded chromosome 7s **(B)** and partial G-banded ideogram **(C)** for case 3. Arrows denote breakpoints.

The MCB pattern on the derivative chromosome 7 showed that the additional material present within the long arm of the abnormal chromosome was a duplication of the region 7q21.2→7q22 (Figure 3).

#### Final karyotype

46,XX,dup(7)(q21.2q22.1)

### Case 4

#### Referral reasons

Age 11 years - mild learning difficulties, hypotonia, lax joints, hyperactive, large head, long face, ?Angelman syndrome.

#### Preliminary karyotype

46,XY,inv(2)(p13p21),t(7;13;18)

#### Investigations

Multiwell whole chromosome paints (WCPs) for every chromosome;

MCB probe sets for chromosomes 13 and 18 (XCyte 13 & XCyte 18 respectively).

**Figure 4:**
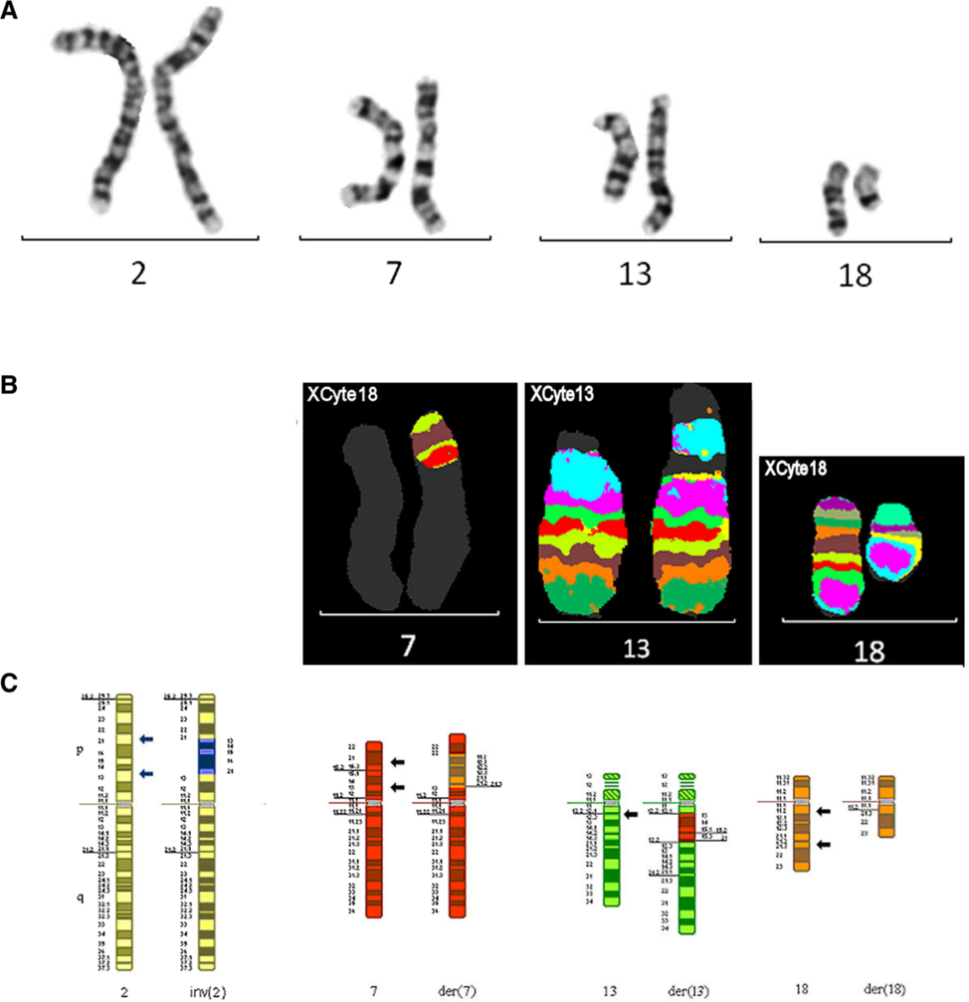
**Chromosomes 2, 7, 13 and 18 for case 4.** G-banded chromosomes 2, 7, 13 and 18 **(A)**, multicolor banded chromosomes 7, 13 and 18 **(B)** and partial G-banded ideogram **(C)** for case 4. Arrows denote breakpoints.

These results together with those of the G-banded chromosome analysis showed that this patient had a paracentric inversion in the short arm of chromosome 2; the proximal two thirds of the chromosome 18 long arm (18q11.2→18q21.3) was directly inserted into the short arm of chromosome 7 and a central segment of the chromosome 7 short arm, comprising about two thirds of the short arm, was inverted and inserted into the chromosome 13 long arm at band 13q12.2 (Figure 4).

#### Final karyotype

46,XY,inv(2)(p13p21),der(7)del(7)(p13p21.2)

ins(7;18)(p13;q11.2q21.3),der(13)  ins(13;7)(q12.2;p13p21.2),der(18)ins(7;18)(p13;q11.2q21.3)

### Case 5

#### Referral reasons

Termination of pregnancy at 23 weeks gestation for ventriculomegaly, large bilateral facial clefts, elongated skull, receding lower jaw, bilateral cleft-lip and palate, pre-auricular skin tags and low-set ears.

#### Preliminary karyotype

46,XX,t(3;12;15)(3qter→3p14.2::15q26.1→15qter;12?p?13.1→12?q?14::12?p?13.1→12?pter;

15pter→15q26.1::3p14.2→3p?ter::12q?14→12qter)

#### Investigations

Subtelomere FISH;

WCP3, WCP12, WCP15;

MCB probe set for chromosome 12 (XCyte 12).

**Figure 5:**
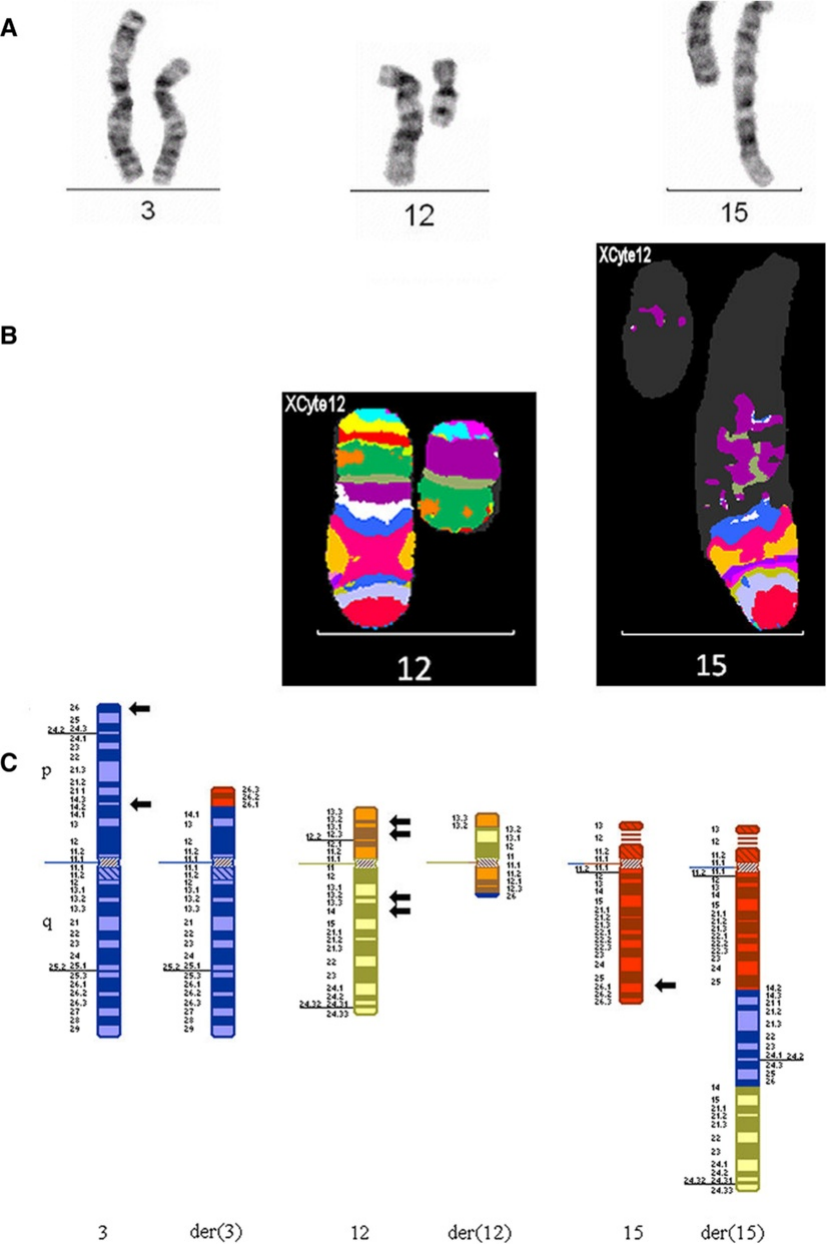
**Chromosomes 3, 12 and 15 for case 5.** G-banded chromosomes 3, 12 and 15 **(A)**, multicolor banded chromosomes 12 and 15 **(B)** and partial G-banded ideogram **(C)** for case 5. Arrows denote breakpoints. Please note that the arms of chromosome 12 are depicted in different colours, for clarity.

The MCB pattern on the derivative chromosome 12 was informative for the band order, and showed monosomy of the region 12p12.3→p13 and the region 12q13.2→12q14 [12] (Figure 5).

#### Final karyotype

46,XX,t(3;12;15)(3qter→3p14.2::15q26.1→15qter;12pter→12p13.2::12q13.?2→12p12.3::3p26.3→

3pter;15pter→15q26.1::3p14.2→3p26.3::12q14→12qter)

### Case 6

#### Referral reasons

Age 3 years - global developmental delay, speech & language delay, poorly formed arches of feet, no obvious dysmorphic features.

#### Preliminary karyotype

46,XX,del(17)(q11.2q25.3),der(22)(22pter→22q13.33::17q?25.3→17q?11.2::17q25.3→17qter)

#### Investigations

Locus-specific FISH; MCB probe set for chromosome 17 (XCyte 17).

The FISH studies showed that probe specific for the long arm subtelomere region of chromosome 17 (D17S928, Abbott) hybridized to the terminal region of the derivative chromosome 22 and to the deleted chromosome 17. The most proximal subtelomere probe for chromosome 22 (D22S1056, Qbiogene (0.74Mb from the telomere)) hybridized to the derivative chromosome 22, while the most distal subtelomere probe (D22S1726, Cytocell (0.097Mb from the telomere)) did not hybridize to the derivative chromosome 22.

**Figure 6:**
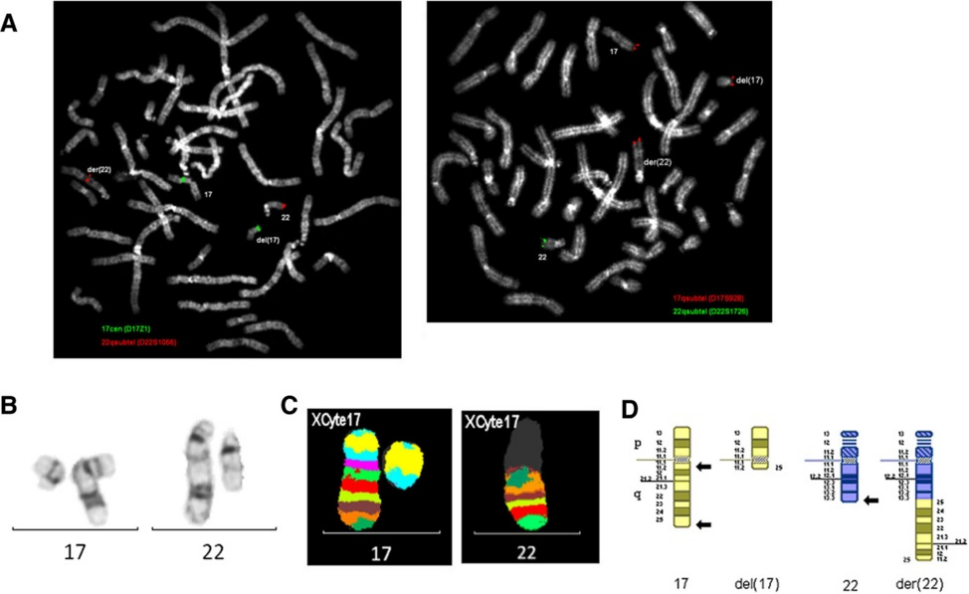
**Chromosomes 17 and 22 for case 6.** FISH images for chromosomes 17 and 22 **(A)**, G-banded chromosomes 17 and 22 **(B)**, multicolor banded chromosomes 17 and 22 **(C)** and partial G-banded ideogram **(D)** for case 6. Arrows denote breakpoints.

The MCB pattern on the derivative chromosome 22 was informative for the band order, and indicated an inversion of the region 17q11.2→q25 (Figure 6).

#### Final karyotype

46,XX,del(17)(q11.2q25.3),der(22)(22pter→22q13.33::17q25.3→17q11.2::17q25.3→17qter)

### Case 7

#### Referral reasons

Age 2 months - CHD – ASD/VSD, triangular face with frontal bossing and suspicion of DiGeorge syndrome.

#### Initial karyotype

46,XY,?dup(11)(p12p13)

#### Investigations

PCP11p, PCP11q;

Multiwell WCPs for all chromosomes;

Microsatellite analysis;

MCB probe set for chromosome 7 (XCyte7).

**Figure 7:**
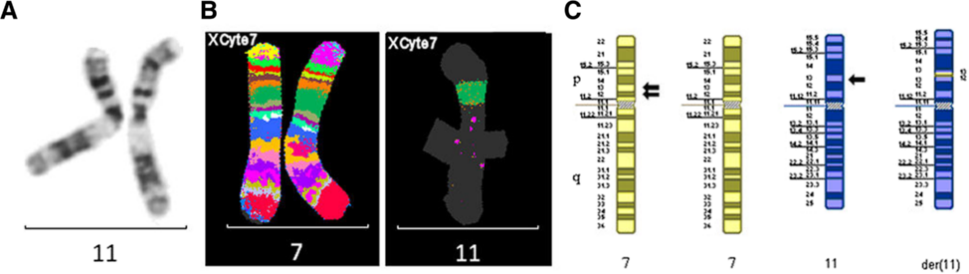
**Chromosomes 7 and 11 for case 7.** G-banded chromosome 11s **(A)**, multicolor banded chromosomes 7 and 11 **(B)** and partial G-banded ideogram **(C)** for case 7. Arrows denote breakpoints.

The MCB pattern showed that the chromosome 7 material present in the proximal region of the short arm of the abnormal chromosome 11 was derived from within the region 7p11.2→7p13. This (7p12.2→7p13) was inserted into the short arm of chromosome 11 at band 11p13. The orientation of the inserted segment of chromosome 7 into chromosome 11 could be determined from the G-banded chromosome pattern (Figure 7).

#### Final karyotype

46,XY,der(11)ins(11;7)(p13;p12.2p13)

### Case 8

#### Referral reasons

Age 3 days - developmental delay, head lag, cleft palate, hypertelorism and left kidney dilation.

Age 6 days - ?mosaicism.

#### Initial karyotype

46,XY (peripheral blood sample)

#### Further investigations due to abnormal phenotype

Array CGH of blood sample: arr 12p11.1p13.33(192,403-34,278,525)x3;

MLPA;

G-banded chromosome analysis of cultured fibroblasts detected a marker chromosome in 82.5% (33/40) of cells examined;

MCB probe set for chromosome 12 (Xcyte12).

The MCB pattern on the marker chromosome was informative for the band order.

**Figure 8:**
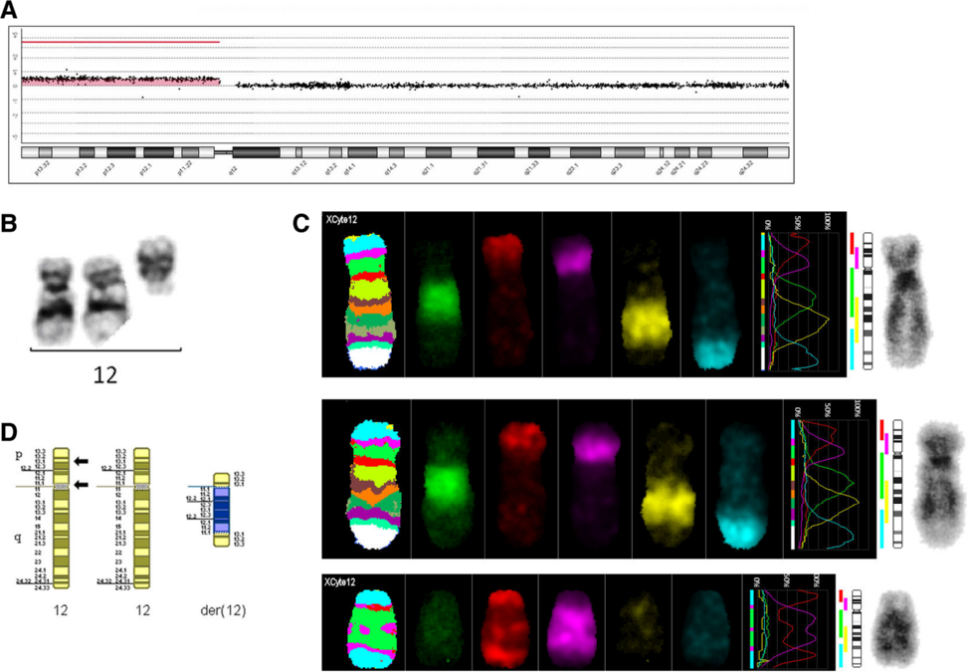
**Chromosome 12s for case 8.** Array CGH trace for chromosome 12 **(A)**, G-banded chromosome 12s **(B)**, multicolor banded fluorochrome profile for chromosome 12s **(C)** and partial G-banded ideogram **(D)** for case 8. Arrows denote breakpoints.

These results confirmed that the marker chromosome was derived from the short arm of chromosome 12, and was not a typical i(12p), but was the result of a breakage/inversion event (with breakpoints at the centromere and at band p13.1) with duplication of the derived product, giving a symmetrical pseudodicentric structure (Figure 8).

#### Final karyotype

47,XY,+der(12)(12pter→12p13.1::12p10→12p13.1::12p13.1→12p10::12p13.1→12pter)[33]/46,XY[7]

## Discussion

Multicolor banding is the most informative hybridization technique for characterization of chromosome rearrangements [12,13]. This is because, compared to WCP probes, MCB can provide specific information regarding the position of breakpoints and imbalance [12,14,15]. This information is invaluable for the clinical management of patients and for characterizing parental balanced chromosome rearrangements as it enables the calculation of accurate reproductive risks for the parents for future pregnancies.

In this laboratory, MCB has been carried out on 8 cases that have undergone either karyotype analysis alone or karyotype analysis and array CGH; in all cases, MCB provided information not available from the other tests carried out. We conclude that MCB is an important adjunct to array CGH and conventional karyotyping; for balanced rearrangements detected by karyotyping, MCB will provide more detailed breakpoint and orientation information, and for duplications detected by array CGH, MCB will detect the genomic location and orientation of the additional material. Multicolor banding therefore still has an important place in modern diagnostic laboratories.

## Conclusions

A modern cytogenetics laboratory should have access to a range of molecular cytogenetic tools, including various in situ hybridisation protocols. Of these, MCB is an important adjunct, which can provide information not available using other techniques.

## Methods

### Karyotyping

For all cases chromosomes were prepared from peripheral blood lymphocyte cultures following standard laboratory procedures. Case 8 also had chromosome preparations made from cultured fibroblasts. Analysis of G-banded chromosomes was carried out at a resolution of 400–550 bands per haploid genome using standard protocols.

### FISH

In situ hybridization studies were carried out for each case using a range of locus specific, chromosome enumeration and subtelomere probes, partial chromosome paints (PCP) and whole chromosome paints (WCP).

### Multicolor banding

Multicolor banding with a cocktail of microdissected region-specific overlapping partial chromosome paints (PCP) was carried out on metaphase chromosomes from peripheral blood samples for cases 1 to 7 and from cultured fibroblasts for case 8, according to the protocol recommended by MetaSystems (GmbH, Altlussheim, Germany). The MCB probes used were specific to the chromosomes involved in the rearrangement and all were from Metasystems GmbH, Altlussheim, Germany. Cells were captured with a CCD camera and processed using the ISIS/mFISH imaging system (Metasystems, Altlussheim, Germany). Fluorescence intensity ratios produced by the region-specific PCPs along the longitudinal axis of the chromosome were used to assign pseudocolours to chromosomal regions by means of the ISIS software.

### MLPA

MLPA was carried out on DNA extracted from peripheral blood for cases 1 and 8 using MLPA kits (P069, P036 or P036B, MRC-Holland, The Netherlands) containing probes for the subtelomere regions of every chromosome, as described previously [16].

### Microsatellite analysis

Microsatellite analysis was carried out on DNA extracted from peripheral blood for cases 3 and 7 using polymorphic microsatellite markers specific for chromosome 7 as described previously [17]. Three markers were informative (D7S820 (7q21.11), D7S796 (7q22.1) and D7S2847 (7q31.31)) for case 3 and two markers specific for the short arm of chromosome 7 (D7S2552 within distal 7p11.2 and D7S628 (7p14.3)) were informative for case 7.

### Array CGH

Array CGH was carried out on DNA extracted from peripheral blood lymphocytes from case 8 as described previously [8], using an oligonucleotide array slide (AMADID 017457; Agilent Technologies, Santa Clara, CA) containing approximately 44,000 probes across the genome.

## Competing interests

The authors declared that they have no competing interests.

## Authors’ contributions

SMB participated in the cytogenetic analysis and interpretation, drafted the manuscript and organized the revisions. AFD participated in the cytogenetic analysis and interpretation and critically revised the manuscript. CMO conceived of the study, participated in its design, helped to draft the manuscript and revised it critically. All authors read and approved the final manuscript.
